# Diagnosis of obstructive sleep apnea with respiratory polygraph in hypercapnic ICU patients

**DOI:** 10.1186/cc14310

**Published:** 2015-03-16

**Authors:** G Gursel, A Zerman, M Aydogdu, B Basarik Aydogan, K Gonderen, S Memmedova, N Sevimli, I Koroglu, Z Isikdogan, M Badoglu, O Ozdedeoglu

**Affiliations:** 1 Gazi University Medical Faculty, Ankara, Turkey

## Introduction

The most frequent reasons for hypercapnic respiratory failure (HRF) in ICUs are COPD and in recent years obesity hypoventilation syndrome (OHS) and obstructive sleep apnea (OSA). Even 15 to 30% of COPD patients also have accompanying OSA. Due to increased upper airway resistance, those patients require higher expiratory pressures (EPAP) during noninvasive ventilation (NIV). In order to prescribe optimal mode and pressures during the ICU stay and at discharge, the intensivist should diagnose the underlying OSA. Portable recording devices have been developed and they were approved at least for the diagnosis in high pretest probability patients with results equal to in-laboratory polysomnography. The aim of this study is to assess whether respiratory polygraph (RPLG) can be used for obtaining diagnostic information of OSA in hypercapnic ICU patients.

## Methods

Patients, with HRF requiring NIV, were included in the study. RPLG studies were conducted under nasal oxygen before NIV, using Philips Respironics Alice PDx® device, which provides the records of pulse oximetry with derived heart rate; snoring and nasal airflow with nasal pressure transducer and nasal thermistor; rib cage, abdominal motion and body position with abdominal and thoracic belts. American Academy of Sleep Medicine 2014 recommendations were used for the diagnosis of OSA and OHS. Because of the diagnostic difficulties of hypopnea in hypoxemic patients, we evaluated only the obstructive apnea index (OAI) instead of the apnea hypopnea index (AHI).

## Results

Thirty-one patients with the mean age of 67 ± 9 years were included in the study. Their mean APACHE II score was 16 ± 5 and BMI was 33 ± 9 kg/m^2^. Admission arterial blood gases were as follows (mean ± SD); pH: 7.33 ± 0.07, PaO_2_: 74 ± 12 mmHg, PaCO_2_: 69 ± 11 mmHg, HCO_3_^-^: 31 ± 5, O_2_Sat%: 92 ± 4. Admission diagnoses of the patients were OHS (36%) and COPD (68%). Mean OAI was 13 ± 6 in patients with OAI >5. Eighty-one percent (*n *= 25) of the recordings were interpretable and clinical and RPLG data supported a new diagnosis of OSA in 14 (56%) patients, and EPAP levels were increased. Laboratory sleep study was recommended to 19% of the patients. At the end of the study 56% of the COPD and 72% of the OHS patients were identified to have OSA.

## Conclusion

Although it underestimates AHI, RPLG is important and technically feasible in ICU patients in suggesting the presence of OSA and in providing information for appropriate NIV management.

